# Comparative Outcomes of Open Simple Prostatectomy in Men with or Without Prior History of Transurethral Resection of the Prostate

**DOI:** 10.5152/tud.2025.24053

**Published:** 2025-07-29

**Authors:** Mohadeseh Ghafouri Ahmadabadi, Kowsar Amirzadeh Gougheri, Alireza Lashay

**Affiliations:** 1 Student Research Committee, Shahid Beheshti University of Medical Sciences School of Medicine, Tehran, Iran; 2 Department of Urology, Shahid Beheshti University of Medical Sciences, Shahid Modarress Hospital, Tehran, Iran

**Keywords:** Prostate, Transurethral resection of the prostate, Surgery, Reoperation, Open simple prostatectomy

## Abstract

**Objective:**

This retrospective cross-sectional study aimed to compare functional and surgical outcomes after open simple prostatectomy (OSP) between patients who underwent prior transurethral resection of the prostate (TURP) and those who did not.

**Methods:**

Between March 2009 and April 2019, 723 patients underwent TURP, of whom 20 (2.7%) subsequently underwent OSP (Group 1). This group was matched with a group of patients who had solely undergone OSP (Group 2), with matching criteria including age, prostate-specific antigen level, prostate volume, and prostate weight.

**Results:**

Group 1 showed a statistically significant lower decrease in hemoglobin levels after surgery (*p = .*006); however, no significant differences were observed between the groups in terms of operation time (*P = .*508), hospital stay (*P = .*065), transfusion rate (*P = .*331), enucleated prostate volume (*P = .*733), or changes in creatinine levels (*P = .*418). Regarding early postoperative complications, the 2 groups showed no significant difference (0.349). Late postoperative complications occurred in 30% of Group 1 and 33% of Group 2, which was not significantly different either (*P* = .241). Both groups achieved similar early continence rates (88%) within the first 6 months after surgery. Late continence rates (after 6 months) were also comparable, with 94% in Group 1 and 88% in Group 2. Finally, no significant differences were found in patient satisfaction levels, measured on a qualitative scale ranging from “dissatisfied” to “highly satisfied.”

**Conclusion:**

Prior TURP did not significantly affect the surgical or functional outcomes of subsequent OSP, with comparable results observed between patients with and without a history of TURP.

## Introduction

Benign prostatic hyperplasia (BPH) is the most common condition affecting the male lower urinary tract. Histologically, it is characterized by the growth of epithelial and stromal tissue within the transition zone of the prostate that anatomically compresses the urethra, causing increased bladder outlet resistance and lower urinary tract symptoms (LUTSs) such as hesitancy, dribbling, incomplete voiding, weak stream, nocturia, frequency, urgency, and urge incontinence.^1^^-^^3^

Currently, the main treatments for BPH are medications and surgery. Surgical options like transurethral resection of the prostate (TURP) and open simple prostatectomy (OSP) are the standard of care and the most effective approaches for the management of BPH when there are complications or LUTS refractory to medical treatment.^4^ Transurethral resection of the prostate is the current standard procedure for men with prostates of 30-80 mL,^5^ whereas OSP is considered the most effective and recommended first-line surgical option for patients with prostate volumes greater than 80 mL, particularly when anatomic endoscopic enucleation of the prostate (AEEP) is unavailable,^6^ or when additional bladder pathologies such as diverticula or stones are present.^7^

About 15-20% of patients who undergo transurethral resection of the prostate for BPH experience recurrent or persistent LUTS and need additional therapy.^8^ One study found that the need for secondary endourological procedures such as TURP, urethrotomy, and bladder neck incision in patients who had previously undergone TURP was 14.7% within 8 years.^9^ Another study showed that the reoperation rate at 8 years was 12.7%.^10^ However, patients are likely to experience recurrent symptoms after re-TURP because the prostate has not been completely removed yet. Open simple prostatectomy may be an option for patients requiring additional therapy for large residual or recurrent adenoma after TURP. However, previous prostatectomy, a small fibrous gland, prior pelvic surgeries and evidence of significant prostate cancer are considered contraindications to simple prostatectomy because they may obstruct access to the prostate gland.^11^ In other words, previous TURP can result in fibrous scarring, changes in tissue layers, and alterations in prostate anatomy, making surgical procedure more challenging.^12^^,^^13^

Heretofore, little has been published on the surgical and functional outcomes of OSP in patients who have previously undergone TURP. In this retrospective study, the authors analyze various perioperative, intraoperative, and postoperative variables associated with simple open prostatectomy in patients with a history of TURP compared to those without a prior TURP. The aim is to evaluate the safety of OSP in patients with a background of earlier TURP.

## Material and Methods

Between March 2009 and April 2019, a total of 723 patients underwent TURP at the authors’ institution. Of these, 20 patients (2.7%) later underwent OSP due to recurrent LUTS (85%), retention (20%), and recurrent hematuria (20%). These patients were designated as Group 1. All patients in Group 1 had significantly enlarged prostates (greater than 70 mL) without diverticula or bladder stones. The authors compared this group with a second group of patients who had only undergone OSP. In Group 2, 2 patients had bladder stones as associated indications for OSP. The 2 groups were matched based on prostate-specific antigen (PSA) levels, preoperative prostate volume by ultrasonography, and postoperative enucleated prostate weight to assess the functional and surgical outcomes between the 2 groups. It is important to note that all OSP procedures at the authors’ institution have been performed using the Freyer technique.

The inclusion criteria included accessibility to patients in order to assess and evaluate their satisfaction rates, postoperative complications, and incontinence rates, unless the patients were lost to follow-up or unreachable by phone. The authors excluded patients who were diagnosed with prostate cancer or had suspicious digital rectal examination before surgery, those who had previous surgery related to urethral stricture, and patients with the history of pelvic radiotherapy. Patients in both groups were comparable considering preoperative parameters including mean age, median level of PSA, and mean prostate volume. The study was approved by the ethical committee of Shahid Beheshti University of Medical science (IR.SBMU.MSP.REC.1398.970). For the present study, informed consent was sought from all patients.

The authors conducted a retrospective cross-sectional evaluation of the surgical and functional outcomes of both groups to compare Group 1, who had a history of TURP, with Group 2, who underwent OSP as their first and only prostate surgery. Early and late complications, patients’ satisfaction rate, and early and late incontinence were assessed.

### Statistical Analysis

To assess the relationships within the data, the authorsemployed statistical analysis using IBM SPSS Statistics, version 25 (IBM SPSS Corp.; Armonk, NY, USA). Chi-square tests were used to analyze categorical variables, while quantitative variables were analyzed using *t*-tests. A *P*-value below .05 was considered statistically significant.

## Results

Both groups were similar regarding preoperative characteristics including age, PSA level, and prostate volume by ultrasonography. The mean ± SD of age, PSA level, and prostate volume were respectively 71.80 ± 8.04 years, 5.79 ± 3.21 ng/mL, and 94.67 ± 49.60 mL in patients with the history of TURP and 73.40 ± 8.56 years, 8.17 ± 8.78 ng/mL, and 108.32 ± 54.71 mL in patients without previous TURP (Table 1).

The mean ± SD interval between previous TURP and open simple prostatectomy was 83.10 ± 48.61 months (min = 12, max = 204 months).

Mean operation time was 112.50 ± 59.35 minutes and 102.50 ± 26.03 minutes respectively, in Group 1 and Group 2 (*P = .*508). The difference between preoperative and postoperative hemoglobin levels was 2.03 ± 1.61 g/dL in patients with previous TURP and 3.45 ± 1.45 g/dL in patients who did not have the history of TURP. In other words, the hemoglobin drop after surgery was significantly lower in Group 1 (*P = .*006). In terms of hospital stay both groups were approximately the same (4.90 ± 2.45 and 3.85 ± 1.04 days; *P = .*065). Changes in creatinine levels after surgery were not statistically different (0.096 ± 0.279 vs. 0.015 ± 0.339 mg/dL; *P = .*418). The transfusion rate was 10% vs. 20% (*P = .*331). In addition, the mean enucleated prostate weight did not significantly differ between 2 groups (62.53 ± 39.58 vs. 67.7 ± 52.97 g; *P = .*733) (Table 2).

In 90% of Group 1 and 75% of Group 2, post OSP histopathology confirmed the diagnosis of BPH. High-grade prostatic intraepithelial neoplasia (HGPIN) was found in 15% of patients in Group 2, and non-specific granulomatous prostatitis (NSGP) was identified in 5% of patients in Group 1. About 5% of patients in both groups had Grade Group 1 adenocarcinoma, while Grade Group 2 adenocarcinoma was found only in 5% of patients in Group 2. Overall, there was no meaningful difference in pathology between the 2 groups (*P = .*260).

The patient satisfaction rate was measured via phone interviews after a mean period of 50 months following the surgery, ranging from very good to very poor, and it did not significantly vary between the 2 groups (*P = .*422). About 83.3% and 11.2% of Group 1 and 72.2% and 5.6% of Group 2 were, respectively, fully satisfied and relatively satisfied. Meanwhile, 5.6% of Group 1 and 11.1% of Group 2 were dissatisfied or had not experienced any improvement after the OSP. Four patients (2from each group) were lost to follow up, so their data were not included in the satisfaction analysis.

Neither the first nor the second group experienced complications during surgery. Early postoperative complications (during hospitalization) were observed in none of the Group 1 members, while 2 patients in Group 2 experienced early complications (1 with massive bleeding 60 minutes after the OSP, which was managed conservatively, and another with a wound infection) (*P = .*349).

The overall late postoperative complication rate, other than incontinence, was 30% in Group 1 and 33% in Group 2. Bladder neck contracture, as a major complication, only occurred in 1 patient in Group 1. Retention was observed in 2 patients in Group 1 and 1 in the second group. Among patients who experienced retention, 2 patients underwent reoperation for OSP due to recurrent retention (one from each group). The reoperations for OSP were performed at 12 months and 15 months after the previous surgery in the first and second groups, respectively, due to recurrent urinary retention caused by regrowth of prostatic adenoma, as confirmed by imaging and urodynamic studies. One patient in the first group was hospitalized due to gross hematuria and clot retention after 22 days. Four patients from the second group returned to us because of refractory LUTS. Urinary tract infection occurred in 2 patients in the first group and 1 in the second group(*P = .*241).

Urinary continence rates were comparable between the TURP group and the non-TURP group. Early incontinence (during the first 6 months after the OSP) was reported in 2 patients in Group 1 (2 with true Urinary incontinence(UI)) and 2 patients in Group 2 (1 with true and 1 with stress UI) (*P = .*721). Regarding late incontinence (after 6 months), 1 patient from the first group complained of stress UI, while 2 patients from the second group were reported to have late UI (1 with stress and 1 with true UI) (*P = .*794) (Table 3). It should be noted that 2 patients from each group were lost to follow-up, and their continence outcomes could not be evaluated.

## Discussion

The effect of a previous TURP on a subsequent open simple prostatectomy is a topic of debate. Despite the development of alternative management options for BPH, OSP remains a viable choice for patients with large prostate glands.^14^ Studies have demonstrated OSP’s effectiveness in achieving long-term positive outcomes, including significant improvements in the International Prostate Symptom Score (IPSS) and post-void residual urine volume, with minimal need for subsequent corrective surgery.^15^

Research indicates that LUTS may recur after TURP.^16^ This highlights the need for a relatively safe interventional treatment to address these recurrent symptoms following TURP. Some men experience recurrent symptoms following TURP that do not respond to medications, and when their prostate size exceeds the threshold for a re-TURP, OSP can become a consideration. However, OSP is not without challenges. Studies have documented increased risks of infection, a higher rate of blood transfusion,^17^ clots retention needing evacuation and bladder neck/urethral contracture,^18^ especially when performed after TURP. This is due to difficulties in accessing prostate tissue and distinguishing between the adenoma and the prostate capsule, which can lead to incomplete resection.^19^^,^^20^

While few studies have directly assessed the functional and surgical outcomes of OSP following TURP, research has been conducted on the outcomes of Radical Prostatectomy (RP) and Laparoscopic Radical Prostatectomy in patients with a history of TURP.^13^^,^^21^^-^^23^ Additionally, studies have explored the need for re-TURP in patients with previous TURP or OSP. For example, Eredics et al^10^ found that 8.3% of patients with a history of TURP and 4.3% of patients with a previous OSP required re-TURP within 8 years.

A recent study by Abedi and colleagues^20^ indicated the feasibility of OSP in patients with a large prostate after previous TURP. However, it was associated with more long-term complications. Given the limited research specifically addressing the outcomes of OSP in patients with a history of TURP, this study aims to contribute to this area.

The authors evaluated the results of OSP in patients with a history of TURP (Group 1) and compared them to patients who had only undergone OSP (Group 2). The authors also assessed differences in incontinence rates and subtypes, surgical complications, and satisfaction levels. While less invasive treatments, such as laparoscopic simple prostatectomy, which offers shorter hospital stays and reduced bleeding for large prostates, and robotic-assisted simple prostatectomy, which overcomes some limitations of laparoscopy, are considered alternatives to OSP,^24^^,^^25^ the lack of access to robotic technology in the authors’ country leads us to prefer OSP for large prostates. It is important to note that the authors did not include erectile dysfunction (ED) in this study as most patients were over 40 years old. ED affects 30–50% of men in this age group and is often multifactorial, commonly related to aging, comorbidities, and lifestyle factors, making it difficult to attribute solely to prostate surgery^26^

This study revealed no significant differences between patients who underwent OSP after TURP and those who had only OSP with respect to several important variables. Specifically, there were no notable differences in postoperative creatinine levels, transfusion rates, length of hospital stay, prostate pathology, or the mean weight of the enucleated prostate. Additionally, rates of early and late incontinence, as well as preoperative, intraoperative, and postoperative complications, and patient satisfaction, revealed no significant differences between the 2 groups. However, hemoglobin drop was lower in Group 1. The lower drop in hemoglobin in Group 1 may be due to the surgeon’s increased precision during the procedure, knowing that they might face a more challenging operation due to the previous TURP. However, further prospective studies are required to explore this finding in more detail and to verify the underlying reasons for the reduced drop in hemoglobin observed in this group. Despite this difference, the overall findings suggest that OSP performed after TURP does not significantly affect the other key outcome measures compared to OSP alone.

Overall, the authors’ findings indicate that OSP may be a viable option following TURP. However, additional research is needed to confirm this assumption.

Open simple prostatectomy after TURP appears to be safe, and a previous TURP does not seem to compromise the surgical or functional outcomes of OSP. However, further studies are needed to confirm these findings.

## Figures and Tables

**Table 1.** Pre-operative Variables

Pre-operative variables	Group 1	Group 2	*P*
Patients (n)	20	20	
Mean age (years)	71.80 ± 8.04	73.40 ± 8.56	.546
Mean total PSA (ng/mL)	5.79 ± 3.21	8.17 ± 8.78	.294
Mean prostate volume (ml)	94.67 ± 49.60	108.32 ± 54.71	.433

**Table 2.** Variables During or After Surgery

Variables during or after surgery	Group 1	Group 2	*P*
Mean operative time (minute)	112.50 ± 59.35	102.50 ± 26.03	.508
Mean hospitalization time (day)	4.90 ± 2.45	3.85 ± 1.04	.065
Transfusion rate after OSP (percent)	10%	20%	.331
Mean enucleated prostate weight (g)	62.53 ± 39.58	67.7 ± 52.97	.733
Mean Hb before surgery (g/dL)	14.17 ± 1.34	13.59 ± 1.79	.572
Mean Hb after surgery (g/dL)	12.14 ± 1.68	10.43 ± 1.62	.002
Mean Cr before surgery (mg/dL)	1.30 ± 0.35	1.33 ± 0.57	.812
Mean Cr after surgery (mg/dL)	1.20 ± 0.20	1.32 ± 0.49	.334

Hb, hemoglobin.

**Table 3.** Postoperation Variables (Number of Patients)

Post-operation variables	Subtype	Group 1	Group 2	*P*
Early incontinence	Stress	0	1	.721
	True	2	1	
	No	16	16	
Persistent incontinence	Stress	1	1	.794
	True	0	1	
	No	17	16	
Early postoperative complications (during hospitalization)	No	20	18	.349
	Wound infection	0	1	
	Hematuria	0	1	
Late postoperative complications (other than incontinence)	No complications	14	12	.241
	Bleeding	1	0	
	Retention	2	1	
	Bladder neck stenosis	1	0	
	UTI	2	1	
	LUTS recurrency	0	4	
Satisfaction	Highly satisfied	15	13	.422
	Semi satisfied	2	1	
	Less satisfied	0	2	
	Dissatisfied	1	2	

Note: Analyses were conducted on patients with complete data available for each specific outcome.

LUTS, lower urinary tract symptom; UTI, urinary tract infection.

## Data Availability

The data that support the findings of this study are available on request from the corresponding author.

## References

[1] Roehrborn CG. Pathology of benign prostatic hyperplasia. Int J Impot Res. 2008;20(S3):S11 S18. (doi: 10.1038/ijir.2008.55) 19002119

[2] Speakman M Kirby R Doyle S Ioannou C. Burden of male lower urinary tract symptoms (LUTS) suggestive of benign prostatic hyperplasia (BPH)–focus on the UK. BJU Int. 2015;115(4):508 519. (doi: 10.1111/bju.12745) 24656222

[3] Kim EH Larson JA Andriole GL. Management of benign prostatic hyperplasia. Annu Rev Med. 2016;67:137 151. (doi: 10.1146/annurev-med-063014-123902) 26331999

[4] Li J Cao D Peng L Comparison between minimally invasive simple prostatectomy and open simple prostatectomy for large prostates: a systematic review and meta-analysis of comparative trials. J Endourol. 2019;33(9):767 776. (doi: 10.1089/end.2019.0306) 31244334

[5] Oelke M Bachmann A Descazeaud A EAU guidelines on the treatment and follow-up of non-neurogenic male lower urinary tract symptoms including benign prostatic obstruction. Eur Urol. 2013;64(1):118 140. (doi: 10.1016/j.eururo.2013.03.004) 23541338

[6] Elterman D Aubé-Peterkin M Evans H UPDATE–Canadian Urological Association guideline: male lower urinary tract symptoms/benign prostatic hyperplasia. Can Urol Assoc J. 2022;16(8):245 256. (doi: 10.5489/cuaj.7906) 35905485 PMC9343161

[7] McVary KT Roehrborn CG Avins AL Update on AUA guideline on the management of benign prostatic hyperplasia. J Urol. 2011;185(5):1793 1803. (doi: 10.1016/j.juro.2011.01.074) 21420124

[8] Seaman EK Jacobs BZ Blaivas JG Kaplan SA. Persistence or recurrence of symptoms after transurethral resection of the prostate: a urodynamic assessment. J Urol. 1994;152(3):935 937. (doi: 10.1016/s0022-5347(17)32614-9) 7519683

[9] Madersbacher S Lackner J Brössner C Reoperation, myocardial infarction and mortality after transurethral and open prostatectomy: a nation-wide, long-term analysis of 23,123 cases. Eur Urol. 2005;47(4):499 504. (doi: 10.1016/j.eururo.2004.12.010) 15774249

[10] Eredics K Wachabauer D Röthlin F Madersbacher S Schauer I. Reoperation rates and mortality after transurethral and open prostatectomy in a long-term nationwide analysis: have we improved over a decade? Urology. 2018;118:152 157. (doi: 10.1016/j.urology.2018.04.032) 29733869

[11] McDougal WS Wein AJ Kavoussi LR Partin AW Peters CA. Campbell-Walsh Urology. 11th ed. Review E-Book: Elsevier Health Sciences; 2015.

[12] Palisaar JR Wenske S Sommerer F Hinkel A Noldus J. Open radical retropubic prostatectomy gives favourable surgical and functional outcomes after transurethral resection of the prostate. BJU Int. 2009;104(5):611 615. (doi: 10.1111/j.1464-410X.2009.08474.x) 19298408

[13] Li H Zhao C Liu P Radical prostatectomy after previous transurethral resection of the prostate: a systematic review and meta-analysis. Transl Androl Urol. 2019;8(6):712 727. (doi: 10.21037/tau.2019.11.13) 32038968 PMC6987598

[14] Suer E Gokce I Yaman O Anafarta K Göğüş O. Open prostatectomy is still a valid option for large prostates: a high-volume, single-center experience. Urology. 2008;72(1):90 94. (doi: 10.1016/j.urology.2008.03.015) 18455772

[15] Varkarakis I Kyriakakis Z Delis A Protogerou V Deliveliotis C. Long-term results of open transvesical prostatectomy from a contemporary series of patients. Urology. 2004;64(2):306 310. (doi: 10.1016/j.urology.2004.03.033) 15302484

[16] Kim SJ Al Hussein Alawamlh O Chughtai B Lee RK. Lower urinary tract symptoms following transurethral resection of prostate. Curr Urol Rep. 2018;19:1 7. 30128964 10.1007/s11934-018-0838-4

[17] Cho JM Moon KT Lee JH Choi JD Kang JY Yoo TK. Open simple prostatectomy and robotic simple prostatectomy for large benign prostatic hyperplasia: comparison of safety and efficacy. Prostate Int. 2021;9(2):101 106. (doi: 10.1016/j.prnil.2020.11.004) 34386453 PMC8322925

[18] Shah AA Gahan JC Sorokin I. Comparison of robot-assisted versus open simple prostatectomy for benign prostatic hyperplasia. Curr Urol Rep. 2018;19(9):71. (doi: 10.1007/s11934-018-0820-1) 29998354

[19] Welliver C Helo S McVary KT. Technique considerations and complication management in transurethral resection of the prostate and photoselective vaporization of the prostate. Transl Androl Urol. 2017;6(4):695 703. (doi: 10.21037/tau.2017.07.30) 28904902 PMC5583058

[20] Abedi AR Allameh F Hojjati SA Ghiasy S Pouri M Montazeri S. The feasibility of open prostatectomy in patients with history of previous prostate surgery. Urol J. 2022;19(2):148 151. (doi: 10.22037/uj.v18i.6468) 35066864

[21] Menard J de la Taille A Hoznek A Laparoscopic radical prostatectomy after transurethral resection of the prostate: surgical and functional outcomes. Urology. 2008;72(3):593 597. (doi: 10.1016/j.urology.2008.03.019) 18762050

[22] Katz R Borkowski T Hoznek A Salomon L Gettman MT Abbou CC. Laparoscopic radical prostatectomy in patients following transurethral resection of the prostate. Urol Int. 2006;77(3):216 221. (doi: 10.1159/000094812) 17033208

[23] Jaffe J Stakhovsky O Cathelineau X Barret E Vallancien G Rozet F. Surgical outcomes for men undergoing laparoscopic radical prostatectomy after transurethral resection of the prostate. J Urol. 2007;178(2):483 487. (doi: 10.1016/j.juro.2007.03.114) 17561162

[24] Patel MN Hemal AK. Robot-assisted laparoscopic simple anatomic prostatectomy. Urol Clin North Am. 2014;41(4):485 492. (doi: 10.1016/j.ucl.2014.07.003) 25306160

[25] Kordan Y Canda AE Köseoğlu E Balbay D Laguna MP de la Rosette J. Robotic-assisted simple prostatectomy: a systematic review. J Clin Med. 2020;9(6):1798. (doi: 10.3390/jcm9061798) PMC7356910 32527020

[26] Khera M Goldstein I. Erectile dysfunction. BMJ Clin Evid. 2011;2011:1803. PMC3217797 21711956

